# ABSENCE OF ANGIOTENSIN TYPE 2 RECEPTOR IS LINKED TO HIGHER BODY WEIGHT AND POOR AEROBIC PERFORMANCE IN AGED MICE

**DOI:** 10.1093/geroni/igae098.3629

**Published:** 2024-12-31

**Authors:** Michael Abadir, Michael Bene, Adam Said, Ruth Marx, Laura Powell, Jeremy Walston

**Affiliations:** University of Maryland, Woodstock, Maryland, United States; Johns Hopkins School of Medicine, Baltimore, Maryland, United States; Emory College of Arts and Sciences, Atlanta, Georgia, United States; Johns Hopkins University, Baltimore, Maryland, United States; Johns Hopkins University, Baltimore, Maryland, United States; Johns Hopkins University, Baltimore, Maryland, United States

## Abstract

Age-related physical decline significantly impacts the quality of life and increases dependence in older adults. The role of angiotensin receptors in the decline of strength and endurance in older organisms has not yet been fully explored. The two main angiotensin receptors are: Angiotensin 1 (AT1) receptor, and Angiotensin 2 (AT2) receptor. This study aimed to investigate the effects of each angiotensin receptor on physical performance in older mice. We compared aged (24 months old) Losartan (an AT1 receptor antagonist) treated wild type (WT) mice to age- and sex-matched mice lacking the AT1 or AT2 receptor (AT1KO or AT2KO). Mice underwent treadmill running until exhaustion or a maximum duration of one hour, with number of falls off belt (NOF), number of gentle stimuli (NGS), and total time on the treadmill belt (TOB) recorded. NGS indicates how often mild stimuli were used to encourage mice to return to the treadmill belt. Body weights showed significant differences between groups by ANOVA (p=0.03), with Tukey HSD indicating AT2KO mice were significantly heavier than WT (p=0.04). TOB analysis indicated significant differences (Kruskal-Wallis, p=0.03), with post-hoc analyses revealing the AT2KO group spent less TOB than WT (p=0.008) and WT los (p=0.01). Differences in NGS were detected, with the AT2KO requiring significantly more stimuli compared to WT (p=0.003), WT los (p=0.001), and AT1KO (p=0.01). These findings highlight the impact of angiotensin receptor modifications on physical performance and suggest that loss of AT2 may result in decreased aerobic performance, potentially due to increased body weight.

